# Up‐regulation of the human‐specific CHRFAM7A gene protects against renal fibrosis in mice with obstructive nephropathy

**DOI:** 10.1111/jcmm.17630

**Published:** 2022-12-07

**Authors:** Bingru Zhou, Yudian Zhang, Xitong Dang, Bowen Li, Hui Wang, Shu Gong, Siwen Li, Fanyin Meng, Juan Xing, Tian Li, Longfei He, Ping Zou, Ying Wan

**Affiliations:** ^1^ Department of Pathophysiology, School of Basic Medical Science Southwest Medical University Luzhou China; ^2^ Institute of Cardiovascular Research, The Key Laboratory of Medical Electrophysiology of Ministry of Education Southwest Medical University Luzhou China; ^3^ Science and Technology Division Southwest Medical University Luzhou China; ^4^ Department of Health Toxicology, Xiangya School of Public Health Central South University Changsha China; ^5^ Indiana Center for Liver Research, Division of Gastroenterology and Hepatology, Department of Medicine Indiana University School of Medicine Indianapolis Indiana USA; ^6^ Richard L. Roudebush VA Medical Center Indianapolis Indiana USA

**Keywords:** CHRFAM7A, epithelial–mesenchymal transition, inflammation, obstructive nephropathy, renal fibrosis, unilateral ureteral obstruction

## Abstract

Renal fibrosis is a major factor in the progression of chronic kidney diseases. Obstructive nephropathy is a common cause of renal fibrosis, which is also accompanied by inflammation. To explore the effect of human‐specific CHRFAM7A expression, an inflammation‐related gene, on renal fibrosis during obstructive nephropathy, we studied CHRFAM7A transgenic mice and wild type mice that underwent unilateral ureteral obstruction (UUO) injury. Transgenic overexpression of CHRFAM7A gene inhibited UUO‐induced renal fibrosis, which was demonstrated by decreased fibrotic gene expression and collagen deposition. Furthermore, kidneys from transgenic mice had reduced TGF‐β1 and Smad2/3 expression following UUO compared with those from wild type mice with UUO. In addition, the overexpression of CHRFAM7A decreased release of inflammatory cytokines in the kidneys of UUO‐injured mice*.* In vitro, the overexpression of CHRFAM7A inhibited TGF‐β1‐induced increase in expression of fibrosis‐related genes in human renal tubular epithelial cells (HK‐2 cells). Additionally, up‐regulated expression of CHRFAM7A in HK‐2 cells decreased TGF‐β1‐induced epithelial‐mesenchymal transition (EMT) and inhibited activation f TGF‐β1/Smad2/3 signalling pathways. Collectively, our findings demonstrate that overexpression of the human‐specific CHRFAM7A gene can reduce UUO‐induced renal fibrosis by inhibiting TGF‐β1/Smad2/3 signalling pathway to reduce inflammatory reactions and EMT of renal tubular epithelial cells.

## 1 INTRODUCTION

Chronic kidney disease (CKD) has become a common human ailment in recent decades, estimated to 8%–16% of the population worldwide.^1^ Common pathological mechanisms for CKD are characterized by progressive nephrotic inflammation and renal interstitial fibrosis^2^ that contributes to the development of end‐stage renal disease (ESRD).^3^ Obstructive nephropathy can be caused by ureteral obstruction and is a common cause of renal fibrosis accompanied by tubular injury and interstitial macrophage infiltration.^4^, ^5^ Renal fibrosis occurs when interstitial fibroblasts proliferate and activate to myofibroblasts that modify and deposit excess extracellular matrix (ECM).^4^

Multiple cellular events and molecular mediators are involved in the development of renal fibrosis, most notably inflammation.^6^ Activated macrophages release pro‐inflammatory factors in response to injury. In the late stages of the proliferative and remodelling phase of wound healing, M1 switches to M2 phenotype and this transition of M1 to M2 phenotype promotes fibrosis by producing cytokines, chemokines and growth factors that affect the behaviour and activation of cells throughout the kidney.^7^, ^8^, ^9^ One such factor is transforming growth factor β (TGF‐β) which is a crucial mediator of tissue fibrosis.^10^ Studies have now demonstrated that TGF‐ β1 signalling through type I TGF‐β receptors leads to phosphorylation and nuclear translocation of Smad2 and Smad 3 that promotes renal fibrosis, while the overexpression of Smad7 prevents TGF‐ β1‐mediated renal fibrosis.^11^, ^12^ Continued activation of Smad‐dependent gene regulatory networks exacerbates the progression of CKD in both experimental animal models and human kidney diseases.^11^, ^12^ TGF‐β also regulates epithelial‐mesenchymal transition (EMT) of certain cell types.^13^ Aberrant TGF‐β signalling and EMT are known to be involved in the pathogenesis of pulmonary fibrosis, renal fibrosis and liver fibrosis.^14^, ^15^ Therefore, effectively inhibiting or reducing the release of pro‐inflammatory and pro‐differentiating factors may provide alternate therapies for the treatment of renal fibrosis.

The human‐specific gene CHRFAM7A is a chimera formed by fusing partial duplication of exons 5–10 of the α7‐N acetylcholine receptor gene (CHRNA7) with the 3′ of a partially duplicated FAM7 gene,^16^, ^17^ which is located on human chromosome 15q13‐q14. The CHRFAM7A gene was first found to be expressed in the central nervous system where it regulates neurotransmitter function and has been associated with the development of mental illness.^18^ However, CHRFAM7A is also expressed in leukocytes and epithelial cells^19^, ^20^ and is involved in the pathology of inflammatory bowel disease,^21^ as well as the cholinergic anti‐inflammatory pathway (CAP).^22^ Previous reports have demonstrated that both humoral factors and CAP can regulate the body's inflammatory response through the vagus nerve.^23^, ^24^ The activation of α7 nicotinic acetylcholine receptors (α7nAChR) in inflammatory cells, such as macrophages, peripheral monocytes, can attenuate the release of pro‐inflammatory cytokines (eg. TNF‐α, IL‐1β, IL‐6) and promote the production of anti‐inflammatory factors such as IL‐10,^25^, ^26^ thereby reducing the body's overall inflammatory response. Several studies have confirmed that activated α7nAChR can effectively alleviate the progression of renal disease in response to acute kidney injury or anti‐glomerular basement membrane glomerulonephritis.^27^, ^28^ Therefore, α7nAChR may provide a new treatment strategy for systemic inflammation and fibrosis during renal injury. Some studies have shown that CHRFAM7A may negatively inhibit the function of α7nAChR/CHRNA7,^29^, ^30^ whereas another study showed altered activity.^31^ Interestingly, more recent studies have confirmed that CHRFAM7A can regulate the anti‐inflammatory effects of activated a7nAChR.^29^, ^32^ Although many changes in the expression of CHRFAM7A in inflammatory bowel disease and Crohn's disease have been documented,^19^, ^21^ little is known about the role of CHRFAM7A in renal injury.

Therefore, the aim of our study was to explore the effect of CHRFAM7A expression on renal fibrosis and inflammation, as well as its involvement in underlying mechanisms of renal damage. We utilized the mouse unilateral ureteral obstruction (UUO) model that has been developed to model kidney injury in humans^33^, ^34^ to study the pathophysiology of renal fibrosis and inflammation during obstructive nephropathy. In this study, we hypothesized that CHRFAM7A would reduce kidney fibrosis through inhibition of the inflammatory response, including macrophage phenotype and alleviation of EMT of renal tubular epithelial cells. Our findings may clarify the role of CHRFAM7A in obstructive nephropathy and facilitate the development of human‐specific gene therapies for renal disease.

## 2 MATERIALS AND METHODS

### 2.1 Materials

Antibodies were purchased from the following companies: Smad‐2/3(D7G7), α‐SMA (#19245), vimentin (#5741) and β‐actin (#P60709) were purchased from Cell Signalling Technology; TGF‐β1 (ab215715) was purchased from Abcam; NF‐κB (10745‐1‐AP) and GAPDH (10494‐1‐AP) were obtained from Pro‐teintech; CHRFAM7A (860647) was purchased from ZEN BIO; Kidney injury molecule 1(KIM‐1) (KCA0319031) antibodies was purchased from R&D system. Recombinant human TGF‐β1 were obtained from Pepro Tech (121809). Lipofectamine 2000 was purchased from Thermo Fisher (11668‐019) and the reverse transcription cDNA synthesis system and SYBR Green were obtained from TIANGEN.

### 2.2 Animal model

CHRFAM7A knock in (CHRFAM7A KI) transgenic mice (C57BL/6 background) were provided by professor Dang at the Cardiovascular Institute in Southwest Medical University. In brief, CHRFAM7A cDNA was a gift from Dr. Andrew Baird (Division of Trauma, Burns and Critical Care, Department of Surgery, University of California San Diego School of Medicine). The cDNA was subcloned into pLVXIRES‐ZsGreen‐1 (Clontech) to produce plasmid pLVX‐IRES‐ZsGreen‐1‐CHRFMA7A as described previously.^20^ Then, the plasmid was digested with HindIII + BamHI, and a 1.9 kb expression cassette containing a CMV promoter and human CHRFAM7A variant 1 (NM_139320.1) ORF were gel‐purified and injected into a zygote. CHRFAM7A‐specific primers were used to confirm presence of the transgene: forward primer, 5′‐CAGTACATCAATGGGCGTGGA‐3′ and reverse primer, 5′‐TGGAATGTGGCGTCAA AGCG‐3′. The PCR product is 405 bp that can be detected by using Mouse Tail Direct PCR Kit (With Dye)‐UNG Kit from FOREGENE.^35^ Wild type (WT) littermates were used as controls. Mice were bred in the SPF system of the Experimental Animal Center of Southwest Medical University. Mice (8–12 weeks old, *n* = 20) were randomly divided into 4 groups: WT mice, CHRFAM KI mice, WT‐UUO mice and CHRFAM7A KI‐UUO mice. CHRFAM7A KI transgenic mice or WT mice underwent a reversible UUO surgery or sham‐surgery procedures. Mice in the UUO surgery group received left ureteral obstruction and the obstruction was lifted after 7 days; mice in the non‐UUO groups underwent a sham surgery. On Day 13, the right ureter was ligated. Mice were sacrificed on Day 16 and kidneys and serum were collected for further analysis. All experimental procedures were approved by the Southwest Medical University Animal Ethics Committee.

### 2.3 Histology

Mouse kidneys were fixed in 4% neutral buffered formalin, paraffin embedded and sectioned at 4 μm using a routine procedure. Haematoxylin–eosin (H&E) staining and Masson's trichrome staining were conducted according to the instructions from the Nanjing Jiancheng.

### 2.4 Measurement of serum creatinine, blood urea nitrogen and interleukin‐6

Concentrations of serum creatinine (Scr) and blood urea nitrogen (BUN) in four groups of mice were determined using kits from Nanjing Jiancheng (C011‐2, C013‐1). Interleukin 6 (IL‐6) was measured using a mouse serum ELISA Quantitation kit, according to the manufacturer's protocol (Elabscience MM‐0163M2).

### 2.5 RNA extraction and quantitative real‐time PCR

Total RNA was extracted from mouse kidney using TRIzol reagent (BioTake Corporation) according to the manufacturer's instruction. Approximately 500 ng total RNA from each sample was used for reverse transcription and cDNA synthesis was performed using TIANGEN reverse transcription kit. PCR primers were designed and synthesized from TSINGKE Biological Technology. cDNA (1 μl/sample) was used for qPCR on a Roche Light Cycler 480. Reactions were performed in a 20 μl reaction mixture containing 10 μl of the 2× SuperReal Premix Plus (SYBR Green, TIANGEN BIOTECH). The sequences of the primers used for real‐time PCR are listed in Table S1. Relative changes in gene expression were calculated using 2^−ΔΔ*C*T^, and all experiments were repeated at least three times.

### 2.6 Western blotting

Protein expression was analysed by Western blot analysis. Mouse kidneys (0.1 g) were lysed in RIPA buffer containing 1 mM PMSF (Beyotime) and fully homogenized. The supernatant obtained by centrifugation was collected as the protein product. After protein concentration were determined by BCA (Beyotimen, P0012S), a certain proportion of SDS was added and samples were boiled. 40 μg of protein was run on 12% SDS‐PAGE gels and transferred onto nitrocellulose membranes. The membranes were incubated overnight at 4°C with primary antibodies against kidney KIM‐1, α‐SMA, TGF‐β1, Smad2/3, vimentin, CHRFAM7A, NF‐κB, GAPDH or β‐actin. The next day, after washing with PBST, the membrane was incubated with a fluorescent secondary antibody (IRDye® 800CW Goat anti Mouse IgG or Goat anti‐Rabbit IgG) for 2 h at room temperature, washed and then scanned using the Odyssey Fc System (LI‐COR, USA). Densitometric analyses were conducted with Image J software.

### 2.7 Cell culture and treatment

Human proximal tubular epithelial cells (HK‐2) were obtained from the Procell Life Science & Technology. HK‐2 cells were cultured on 10 cm Petri dishes in DMEM with a mixture of 10% foetal bovine serum, 50 U/ml penicillin and 50 mg/ml streptomycin and incubated at 37°C in a humid atmosphere incubator with 95% O_2_ and 5% CO_2_. HK‐2 cells were divided into 6 groups: group A, HK‐2 cells basal control; group B, HK‐2 cells with TGF‐β1 (Pepro Tech) stimulation; group C, HK‐2 cells with transfection of empty vector plasmid; group D, HK‐2 cells with transfection of empty vector plasmid and TGF‐β1 stimulation; group E, HK‐2 cells with transfection of CHRFAM7A plasmid; group F, HK‐2 cells with transfection of CHRFAM7A plasmid and TGF‐β1 stimulation. The corresponding plasmids (empty vector or CHRFAM7A) were transfected into HK‐2 cells in four groups (C, D, E and F), respectively. After 24 hours of transfection, the cells in three groups (B, D and F) were stimulated with TGF‐β1 at a dose of 20 ng/ml for 24 h, after which the six groups of cells were collected. q‐PCR was used to detect mRNA expression of CHRFAM7A, fibronectin (FN‐1), α‐SMA, E‐cadherin, N‐cadherin and vimentin in the above six groups of HK‐2 cells. Western‐blot was used to measure protein expression of TGF‐β1, Smad2/3 and vimentin in these cells.

### 2.8 Statistical analysis

All values were expressed as mean ± SD. Statistical differences of the data were determined using One‐Way anova (GraphPad Prism 8). Differences were considered as statistically significant when *p* value <0.05.

## 3 RESULTS

### 3.1 Expression of CHRFAM7A in kidneys of transgenic mice

**FIGURE 1:**
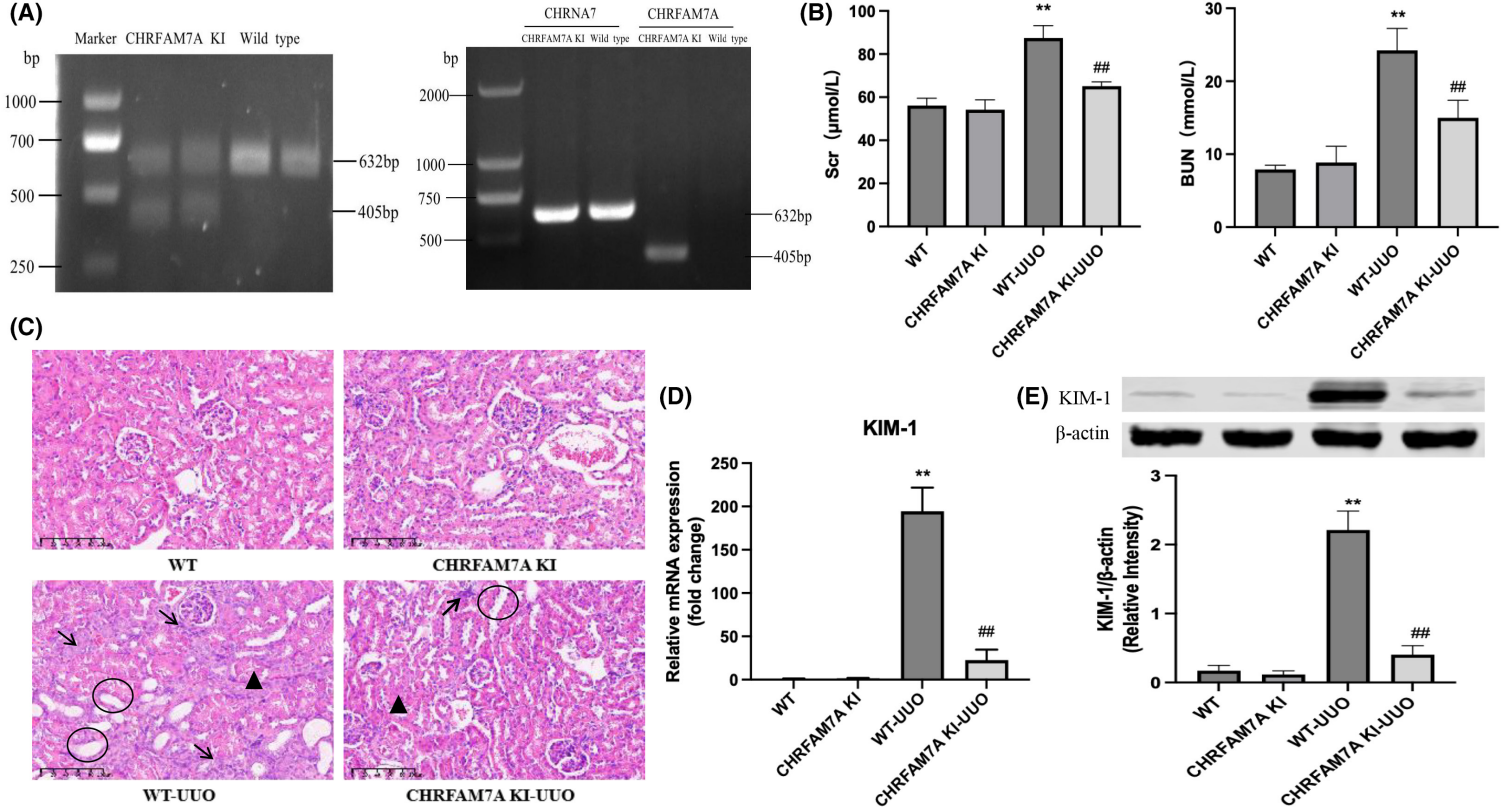
Overexpression of CHRFAM7A reduces renal injury in mice with unilateral ureteral obstruction (UUO). (A) The CHRFAM7A gene was expressed only in mice of the CHRFAM7A KI group, but not in mice of the WT group. CHRFAM7A gene was confirmed to be expressed in kidneys of CHRFAM7A KI mice, but not in the kidneys of WT mice. (B) Detection of serum creatinine (Scr) and blood urea nitrogen (BUN) levels in mice (*n* = 5). (C) H&E staining in the kidneys from the four groups of mice (×200). Arrows indicate inflammatory cells, circles indicate renal tubular dilation and triangles indicate edema of renal tubular epithelial cells. Scale bar = 100 μm. (D, E) Detection of KIM‐1 mRNA expression by q‐PCR and protein expression by Western‐blot in kidneys from the indicated groups of mice (*n* = 5). **p < 0.01 vs. WT group; ^##^ *p* < 0.01 vs. WT‐UUO group. CHRFAM7A KI, CHRFAM7A knock in; WT, wild type.

First, we verified that CHRFAM7A is expressed in the kidneys of transgenic mice. We crossed WT C57BL/6 female mice with male mice that express human‐specific CHRFAM7A (CHRFAM7A KI) and bred offspring carrying the transgene with WT mice to obtain the F4 generation mice. We then performed PCR‐agarose gel electrophoresis on kidney tissue of WT and transgenic mice. The results showed that CHRFAM7A KI mice not only had a 632 bp fragment of CHRNA7 DNA, as expected, but also had a 405 bp fragment of CHRFAM7A. In addition, CHRFAM7A was also expressed in the kidneys of transgenic mice (Figure 1A and Figure S2A). Due to the overexpression of CHRFAM7A, the expression of CHRNA7 was down‐regulated in the mouse kidney (Figure S1A).

### 3.2 Overexpression of CHRFAM7A reduces renal function and structural kidney damage in UUO mice

To investigate the role of CHRFAM7A in the kidney after UUO injury, mice were randomly divided into the following groups: WT, CHRFAM7A KI, WT‐UUO and CHRFAM7A KI‐UUO. As shown in Figure 1B, the levels of Scr and BUN were significantly higher in the WT‐UUO group than WT group. However, Scr and BUN levels in the CHRFAM7A KI‐UUO group were obviously reduced when compared with WT‐UUO group (Figure 1B).

As shown in Figure 1C, the size and shape of the glomeruli were normal in WT mice, and there was no obvious hyperaemia and inflammatory cell infiltration in the renal stroma. Compared with the WT group, the kidneys from WT‐UUO mice showed obvious hydronephrosis and dilation of the renal pelvis and calices with varying degrees of renal tubular dilatation, oedema of renal tubular epithelial cells and infiltration of inflammatory cells in the renal stroma interstitium (Figure 1C). Interestingly, these pathologic changes were not as pronounced or absent in kidneys from CHRFAM7A KI‐UUO mice compared with the WT‐UUO mice (Figure 1C).

We then examined the expression of KIM‐1 in mouse kidney tissues, which is usually detected earlier than traditional indicators of kidney damage, such as Scr and BUN, and can be used for early detection of renal injury. Therefore, we measured mRNA and protein expression of KIM‐1 in WT and transgenic mouse kidneys to make a preliminary assessment of renal injury. As shown in Figure 1D,E, the mRNA and protein expression of KIM‐1 in the kidney of WT‐UUO mice was significantly increased compared with the WT group. However, CHRFAM7A KI‐UUO mice expressed significantly less KIM‐1 than WT‐UUO mice (Figure 1D,E). Together, these results suggested that the human‐specific CHRFAM7A gene may be involved in the protection of renal function and structure in obstructive nephropathy.

### 3.3 The expression of CHRFAM7A reduces the inflammatory response after UUO

**FIGURE 2:**
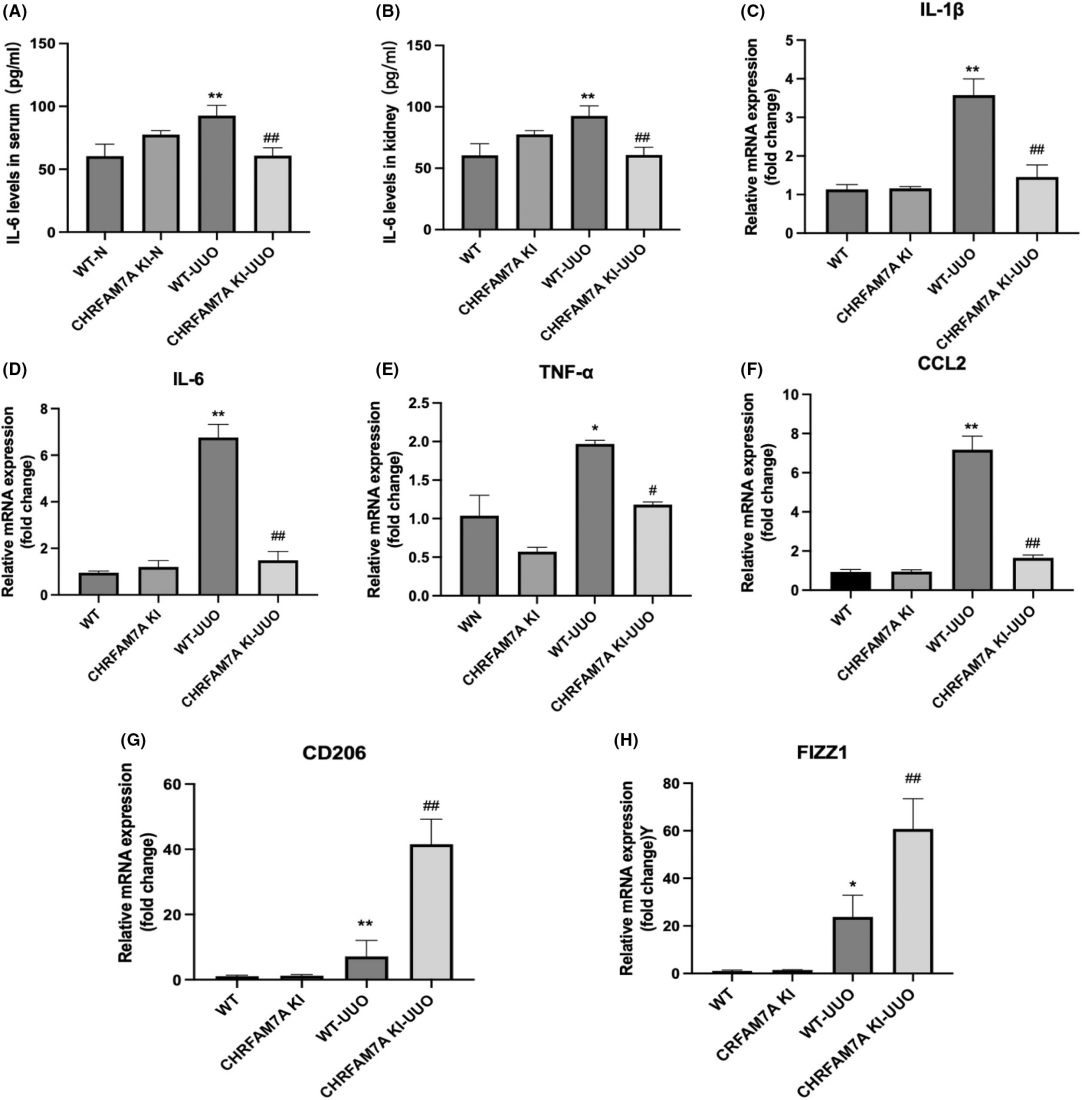
Overexpression of CHRFAM7A reduces unilateral ureteral obstruction (UUO)‐induced inflammatory cytokines and grow factors. (A, B) IL‐6 levels in serum and kidney were measured by ELISA (*n* = 5). All values are expressed as mean ± SD. (C–H) The expression of inflammatory factors IL‐1β, IL‐6, TNF‐α, CCL2, CD206 and FIZZ1in mouse kidney was detected by q‐PCR. Up‐regulation of CHRFAM7A reduced mRNA expression of IL‐1β, IL‐6, TNF‐α and CCL2 in response to UUO mice but increased the expression of CD206 and FIZZ1 compared with WT‐UUO mice (*n* = 5). **p < 0.01 vs. WT group; ^#^ *p* < 0.05, ^##^ *p* < 0.01 vs. WT‐UUO group.

It has been reported that the human specific CHRFAM7A gene plays a key role in the regulation of inflammation.^29^, ^30^, ^31^ To determine whether CHRFAM7A can influence the renal inflammatory response after UUO injury, we measured the mRNA expression of IL‐1β, IL‐6, TNF‐α and CCL2 (factors secreted by M1 phenotype macrophage) in mouse kidney tissues. While the expression of these genes was expectedly higher in the kidneys of WT‐UUO mice than that in WT mice kidneys, they were significantly reduced in CHRFAM7A KI‐UUO mice compared with WT‐UUO mice (Figure 2C–F). Our results revealed that the expression of CHRFAM7A may reduce the release of inflammatory factors in the kidney following UUO injury. Furthermore, while the expression of markers for anti‐inflammatory M2 macrophage (CD206 and FIZZ1) was increased in WT‐UUO mice compared with WT mice, they were much more potentiated in CHRFAM7A KI‐UUO mice than in WT‐UUO mice (Figure 2G,H).

In addition, we measured inflammatory cytokine IL‐6 concentration in the serum and kidneys in the four groups of mice. As shown in Figure 2A,B, IL‐6 concentration circulating in serum and found in the kidneys were expectedly increased in WT‐UUO mice compared with WT mice. However, IL‐6 levels in both circulation and within the kidney were significantly reduced in CHRFAM7A KI‐UUO mice compared with WT‐UUO mice (Figure 2A,B). Furthermore, the overexpression of the human specific CHRFAM7A gene reduced the protein expression of NF‐κB in UUO mice (Figure S2B). Therefore, these results suggested that CHRFAM7A may inhibit the UUO‐induced renal inflammatory response.

### 3.4 Overexpression of CHRFAM7A inhibits renal fibrosis following UUO in mice

**FIGURE 3:**
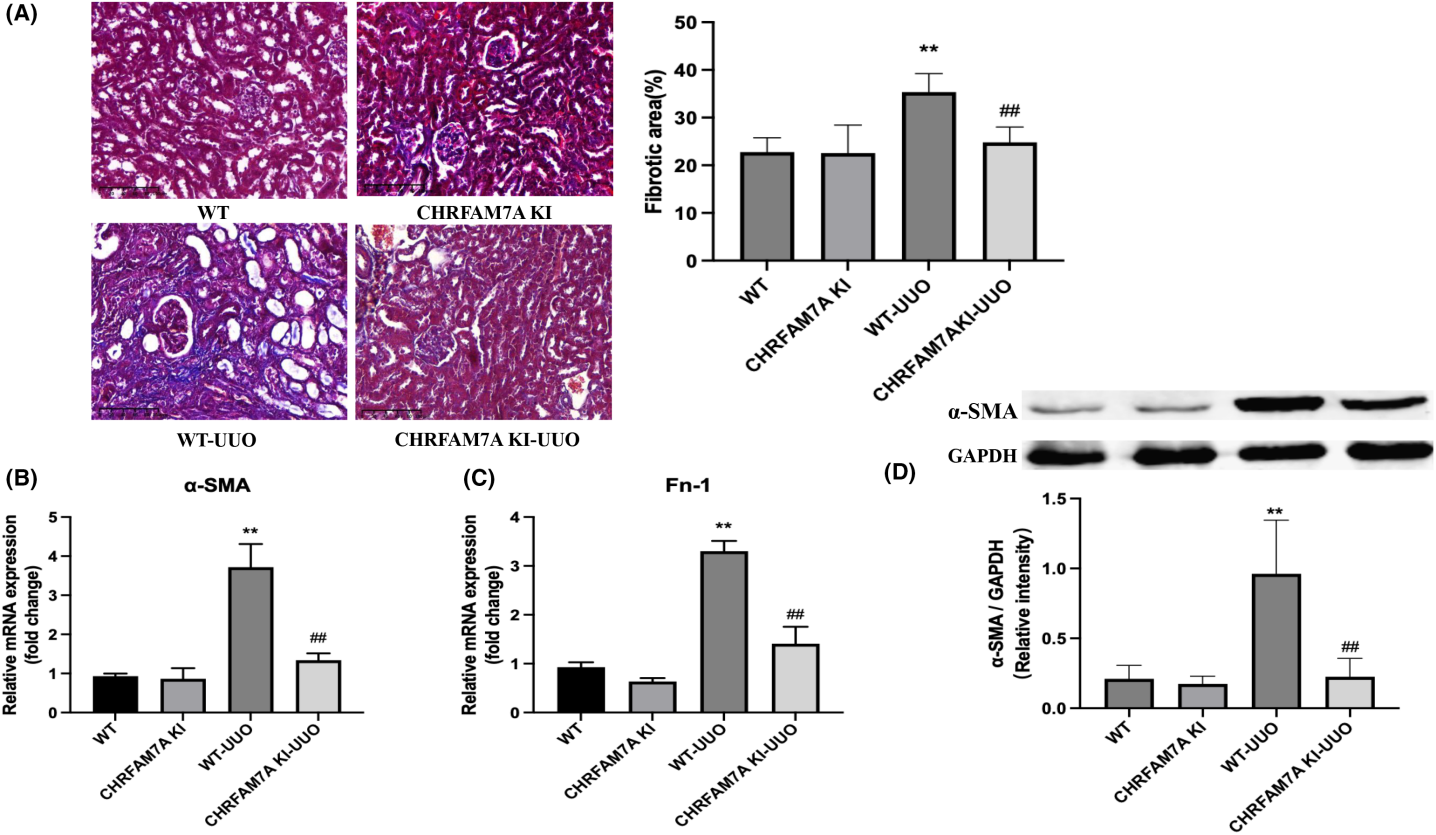
Overexpression of CHRFAM7A delays the progression of renal fibrosis in mice after unilateral ureteral obstruction (UUO). (A]) Representative images and quantitation of Masson's trichrome staining in kidneys from the indicated groups of mice (×200). Scale bar = 100 μm. (B, C) The expression of α‐SMA and fibronectin (FN‐1) in kidneys was detected by q‐PCR. The overexpression of CHRFAM7A reduced the mRNA expression of these fibrotic factors in the kidneys of UUO mice (*n* = 5). WT‐UUO group. (D) Western blot was used to detect the protein expression of α‐SMA in kidney tissues from indicated groups of mice (*n* = 4). Semi‐quantitative analysis of α‐SMA for the Western blot data is shown. **p < 0.01 vs. WT group; ^##^ *p* < 0.01 vs. WT‐UUO group.

To determine the effects of the human specific CHRFAM7A gene on the development of renal fibrosis following UUO in mice, we examined morphological changes in kidney tissue and collagen deposition by staining paraffin sections with Masson's trichrome. Representative images are shown in Figure 3A. No abnormal tubular morphology was observed in WT or CHRFAM7A KI groups; a small amount of collagen (blue staining) was present around tubules, glomeruli and in the renal interstitium. Kidneys from WT‐UUO mice had dense interstitial fibrosis compared with WT mice, confirming obvious UUO‐induced renal fibrosis. But in CHRFAM7A KI‐UUO mice, the fibrotic area was smaller than that of WT‐UUO mice (Figure 3A).

We also determined gene expression of fibrosis markers α‐SMA and fibronectin (FN‐1) in mouse kidneys from all four group. We also determined protein levels of α‐SMA in kidneys from each group were measured by Western blot. The mRNA expression and protein level of above fibrotic markers in the kidneys from the WT‐UUO group of mice were significantly increased compared with the WT mice. While the CHRFAM7A KI‐UUO mice displayed less mRNA and protein expression of these fibrotic markers compared with WT‐UUO mice (Figure 3B–D). These results suggested that the up‐regulation of CHRFAM7A can alleviate UUO‐induced renal fibrosis.

### 3.5 The expression of CHRFAM7A inhibits TGF‐β1/Smad2/3 signalling pathway in kidneys after UUO injury

**FIGURE 4:**
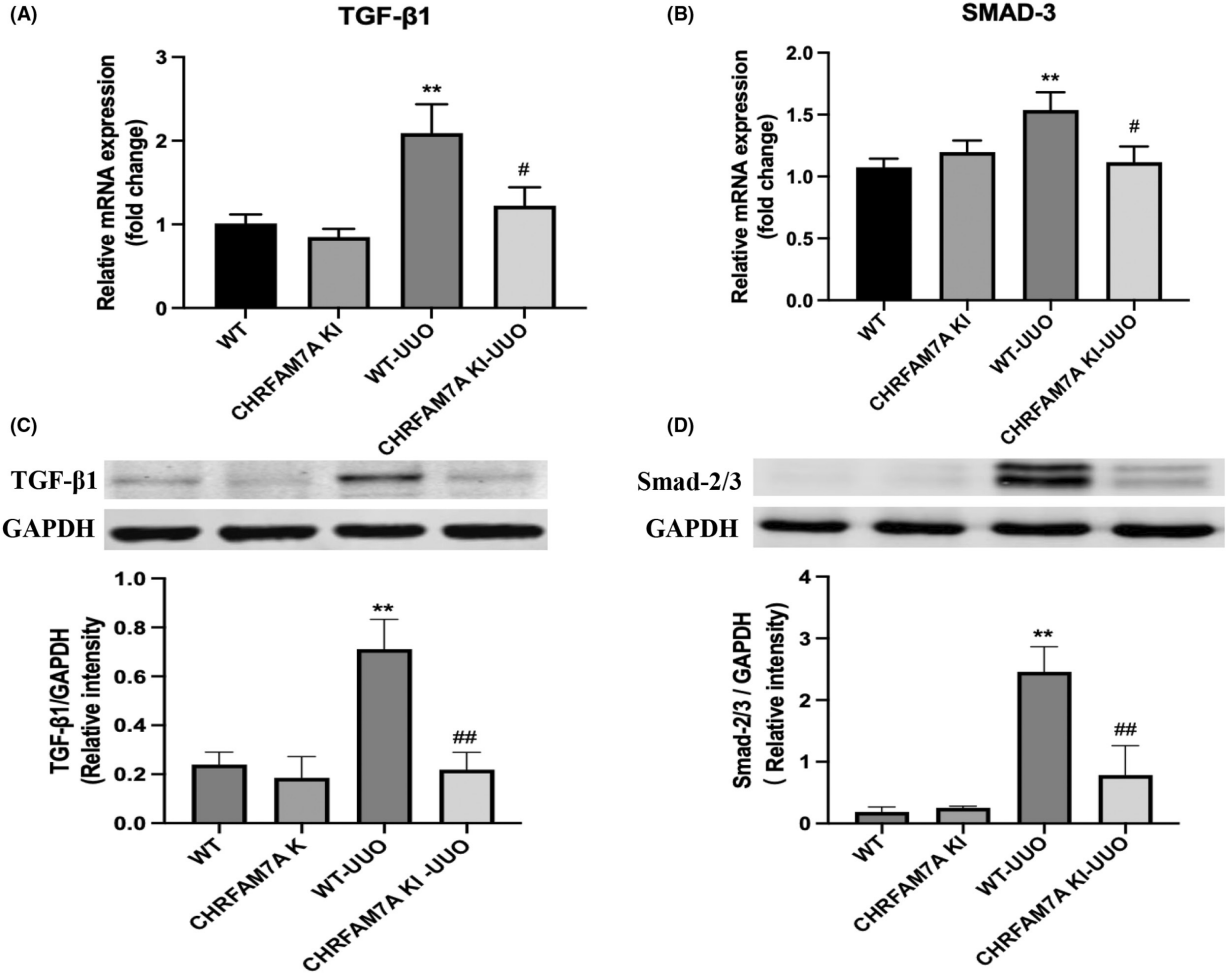
Overexpression of the CHRFAM7A gene inhibits UUO‐induced TGF‐β1/Smad2/3 signalling pathway activation. (A, B) mRNA expression of TGF‐β1 and Smad3 in mouse kidney tissues was detected by q‐PCR. The high expression of CHRFAM7A reduced gene expression of those factors in the kidneys of UUO‐injured mice (*n* = 5). (C, D) Western‐blot was used to detect the protein expression of TGF‐β1 and Smad2/3 in kidney tissues from above four groups of mice; densitometric analysis of the Western blot data for these factors was shown. *p < 0.05, **p < 0.01 vs. WT group; ^#^ *p* < 0.05, ^##^ *p* < 0.01 vs. WT‐UUO group.

Next, in order to explore mechanisms by which CHRFAM7A expression can alleviate UUO‐induced renal fibrosis, we determined mRNA and protein expression of TGF‐β1 and Smad2/3 in kidneys from each group. We observed that TGF‐β1 and Smad2/3 expression in the kidneys from WT‐UUO mice was significantly increased compared with WT mice, but not in the CHRFAM7A KI‐UUO mice (Figure 4A–D). Our data imply that CHRFAM7A expression may alleviate renal fibrosis in UUO mice through inhibiting the TGF‐β1/Smad2/3 signalling pathway.

### 3.6 Overexpression of CHRFAM7A decreases the expression of fibrosis markers in HK‐2 cells stimulated by TGF‐β1

**FIGURE 5:**
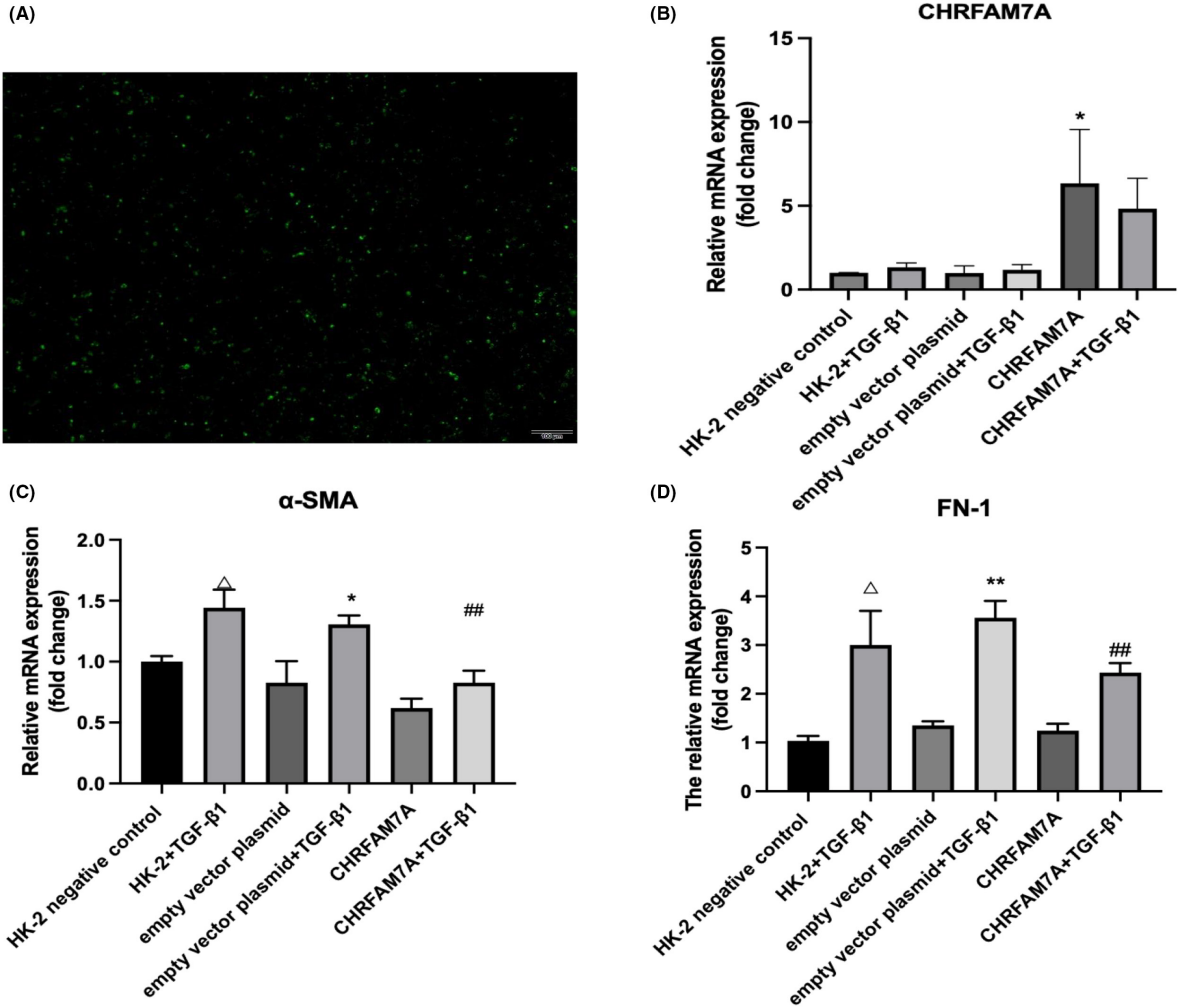
Overexpression of CHRFAM7A decreases expression of fibrotic markers stimulated by recombinant human TGF‐β1 in HK‐2 cells. (A) Fluorescence images of HK‐2 cells with transfection of pLVX‐GFP‐CHRFAM7A plasmid. Scale bar = 100 μm. (B) Human‐specific CHRFAM7A is highly expressed in HK‐2 cells transfected with the CHRFAM7A plasmid. (C, D) q‐PCR results showed that overexpression of the human‐specific CHRFAM7A gene reduced TGF‐β1‐induced increases of fibrotic markers α‐SMA and fibronectin in HK‐2 cells. ^Δ^ *p* < 0.05 vs. HK‐2 negative control; *p < 0.05, **p < 0.01 vs. empty vector; ^#^ *p* < 0.05, ^##^ *p* < 0.01, vs. empty vector + TGF‐β1 group.

Since the findings in vivo showed that the human specific CHRFAM7A gene may have a protective effect on the kidneys following UUO injury in mice, we next utilized in vitro cell culture of human renal tubular epithelial cells (HK‐2) to explore the underlying mechanisms that may be involved. HK‐2 cells were transfected with a CHRFAM7A plasmid or empty vector plasmid for 24 hours, and then stimulated by recombinant human TGF‐β1 for 24 hours to induce EMT. Most of the transfected cells expressed green fluorescent protein (GFP; Figure 5A), reflecting the transfection was successful. We measured the mRNA expression of CHRFAM7A in six groups of HK‐2 cells by q‐PCR. Compared with empty vector control groups, the mRNA expression of CHRFAM7A in the CHRFAM7A group and CHRFAM7A + TGF‐β1 stimulation group was significantly increased (Figure 5B). The expression of CHRNA7 was not much different among the six groups of HK‐2 cells (Figure S1B). Furthermore, the mRNA expression of fibrotic markers α‐SMA and FN‐1 induced by TGF‐β1 treatment was reduced in HK‐2 cells that overexpressed CHRFAM7A (Figure 5C,D).

### 3.7 The overexpression of CHRFAM7A inhibits TGF‐β1‐induced epithelial‐mesenchymal transition in HK‐2 cells

**FIGURE 6:**
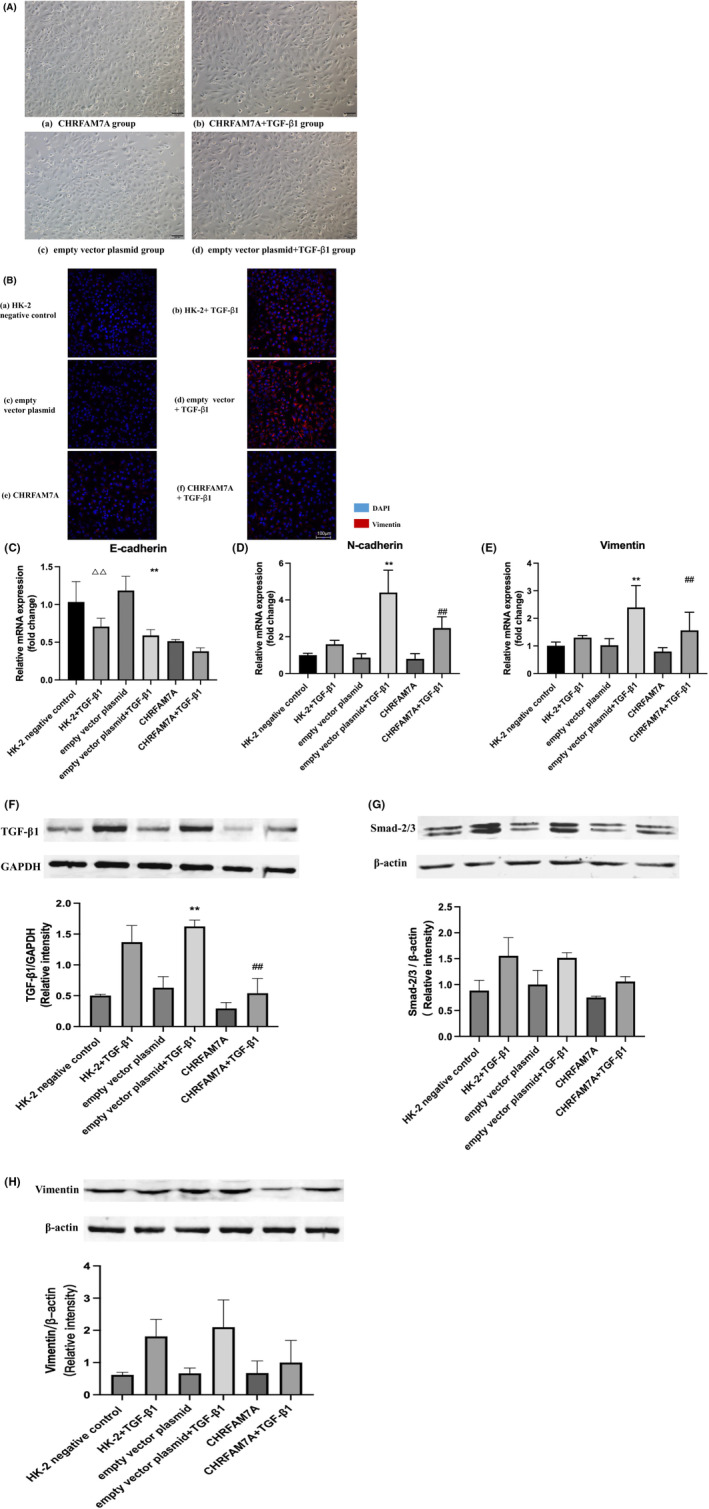
Overexpression of CHRFAM7A inhibits TGF‐β1‐induced epithelial‐mesenchymal transition of HK‐2 cells. (A) Representative brightfield images demonstrate morphological changes seen in HK‐2 cells treated with TGF‐β1 after transfection of human specific CHRFAM7A plasmid or empty vector (×100). (a) HK‐2 cells were transfected with CHRFAM7A plasmid. (b) HK‐2 cells were transfected with CHRFAM7A plasmid, followed by TGF‐β1 stimulation (20 ng/ml) for 24 h. (c) HK‐2 cells were transfected with empty plasmid, (d) HK‐2 cells were transfected with empty plasmids, followed by TGF‐β1 TGF‐β1stimulation (20 ng/ml) for 24 h. (B) Immunofluorescent staining for mesenchymal marker vimentin in HK‐2 cells. Scale bar = 100 μm. (C–E) q‐PCR results indicated that the overexpression of CHRFAM7A inhibited the TGF‐β1‐induced increase in of N‐cadherin and vimentin in HK‐2 cells. (F–H) Western blot was used to detect the protein expression of TGF‐β1, Smad2/3 and vimentin in HK‐2 cells from the indicated groups. The overexpression of CHRFAM7A inhibited TGF‐β1‐induced expression of TGF‐β1, Smad2/3 and vimentin in HK‐2 cells. ^ΔΔ^ *p* < 0.01 vs. HK‐2 negative control; **p < 0.01 vs. empty vector; ^##^ *p* < 0.01, vs. empty vector + TGF‐β1 group.

Next, we measured the gene and protein expression of the factors related to trans‐differentiation in above groups of HK‐2 cells. After 24 h of treatment with TGF‐β1, the cobblestone‐like epithelium of HK‐2 cells changed into the shape of spindle fibroblasts (Figure 6A). Scratch test showed that the overexpression of CHRFAM7A inhibited the migration movement of HK‐2 cells stimulated with TGF‐β1 (Figure 2C,D). Immunofluorescent staining demonstrated that an increase in vimentin expression in HK‐2 cells treated with TGF‐β1. However, the overexpression of CHRFAM7A partly reversed this change (Figure 6B). Gene expression of epithelial cell marker E‐cadherin in HK‐2 + TGF‐β1 group and empty vector + TGF‐β1 group was significantly decreased compared with their respective control group (Figure 6C). The mRNA expression of mesenchymal cell markers N‐cadherin and vimentin in the empty vector + TGF‐β1 group was significantly increased compared with empty vector group. The expression of these markers in the CHRFAM7A + TGF‐β1 group was also significantly increased compared with CHRFAM7A group, however, they were still significantly less than what was observed in the empty vector + TGF‐β1 group (Figure 6D,E).

Furthermore, we examined the protein expression of TGF‐β1 and Smad2/3 in HK‐2 cells in response to CHRFAM7A overexpression and TGF‐β1 treatment. The findings indicated that protein levels of TGF‐β1 in the empty vector plasmid + TGF‐β1 group were obviously increased compared with empty vector plasmid group. The cells in CHRFAM7A + TGF‐β1 group also showed increased expression of these two proteins compared with the CHRFAM7A group. But the protein levels of TGF‐β1 and Smad2/3 in CHRFAM7A + TGF‐β1 group were markedly decreased compared with the empty vector plasmid + TGF‐β1 group. In these groups, changes in vimentin were consistent with the changes in TGF‐β1 and Smad‐2/3 (Figure 6F–H). Our findings suggested that the overexpression of the human‐specific CHRFAM7A gene can inhibit TGF‐β1‐induced EMT in HK‐2 cells and TGF‐β1/Smad2/3 signal axis, thus, reduce renal fibrosis that develops in response to obstructive nephropathy.

## 4 DISCUSSION

Chronic kidney disease has become a serious threat to human health, and inflammation and fibrosis has an important role in its development.^6^ Therefore, it is important to therapeutically prohibit renal inflammation and fibrosis in order to slow down the progression of CKD. In the current study, we revealed that expression of human‐specific CHRFAM7A can alleviate kidney damage in mice in response to an obstructive injury (UUO). Specifically, H&E and Masson's trichrome staining revealed less injury‐induced renal interstitial oedema and deceased fibrosis in CHRFAM7A overexpression transgenic mice compared to WT mice. We also detected alleviation of renal injury markers and inflammatory cytokines present within the kidney after UUO in CHRFAM7A mice. Our results suggest that human‐specific CHRFAM7A may be a viable target to alleviate kidney injury, specifically the progression of fibrosis that leads to CKD.

It is noteworthy that inflammation plays a crucial role in the initiation and progression of renal fibrosis.^36^ To explore whether the effect of CHRFAM7A on UUO‐induced renal fibrosis was related to the regulation of inflammatory signalling pathways, we measured the mRNA expression of inflammatory factors such as IL‐1β, IL‐6, TNF‐α and CCL2 in kidneys, as well as IL‐6 protein concentration in the serum and kidney. Our study showed that mRNA and protein expression of the inflammatory factors listed above were significantly reduced in kidneys from CHRFAM7A KI‐UUO mice compared with those from WT‐UUO mice, suggesting that human‐specific CHRFAM7A gene can inhibit the release of certain inflammatory cytokines and reduce the inflammatory response caused by UUO. It has been previously reported that the two subunits of CHRNA7 and CHRFAM7A are highly homologous, and their overall structure is similar, which may indicate that there may be a certain relationship between the two subunits.^16^ Annalisa et al. demonstrated that primary macrophages that simultaneously expressed CHRNA7 and CHRFAM7A had an increased anti‐inflammatory response after stimulation with lipopolysaccharide (LPS) when compared with macrophages that expressed only one of the genes.^22^ In addition, Zhang et al. observed that the CHRFAM7A gene could inhibit p38/JNK signalling and down‐regulate inflammation and oxidative stress caused by radiation therapy.^37^

We also investigated the expression of markers for non‐activated M2 macrophages, CD206 and FIZZ1. As mentioned above, the overexpression of CHRFAM7A inhibited M1 macrophage activation reflected by decreased expression of M1 macrophage markers such as IL‐6, IL‐1β, TNF‐α and CCL2 (also known as monocyte chemoattractant protein‐1 [MCP‐1]) in response to UUO compared with WT mice. Meanwhile, the expression of M2 macrophage markers such as CD206 and FIZZ1 was increased after UUO in mice that overexpressed CHRFAM7A, suggesting an increased macrophage transition from M1 to M2 phenotype in these mice. It has been reported that the overexpression of CHRFAM7A may attenuate cerebral ischaemia–reperfusion injury through promoting microglia (the resident macrophage in the central nervous system) polarization to M2 phenotype.^38^ Our data consistently observed a decreased M1 pro‐inflammatory macrophage phenotype and increased anti‐inflammatory M2 phenotype, which may reduce injury caused by inflammatory cells and factors at the early stage of injury, thus alleviating the development of UUO‐induced renal fibrosis. Furthermore, Costantini speculated that increased CHRFAM7A expression results in the differentiation of macrophages that are polarized to an anti‐inflammatory phenotype and could explain how CHRFAM7A expression improved mouse resiliency after burn injury.^39^ Taken together with the current findings, we can speculate that CHRFAM7A may attenuate the inflammatory response after renal injury through regulating the α7nAChR‐mediated cholinergic anti‐inflammatory pathway.

Our results also demonstrated that mRNA and protein levels of fibrosis‐related factors in the kidney of the CHRFAM7A KI‐UUO mice were significantly lower than those in WT‐UUO mice, suggesting that CHRFAM7A expression can down‐regulate the expression of fibrotic factors in the kidney following injury. Renal injury is accompanied by infiltration and activation of different types of inflammatory cells and secretion of inflammatory factors, including chemokines, interleukins and tumour necrosis factors that lead to organ fibrosis.^6^ In turn, fibrotic signalling can lead to further activation of immune cells, forming a vicious cycle, which promotes tissue damage and renal fibrosis. For example, when the kidneys were damaged, renal tubular epithelial cells can release inflammatory chemokine CCL2, contributing to an influx of monocytes, T cells and fibrocytes.^40^ Monocytes differentiate into M1 or M2 macrophages. M1 macrophages produce pro‐inflammatory cytokines, such as IL‐1β, IL‐6 and M2 macrophages produce TGF‐β, which accelerates inflammation and fibrosis.^41^ After UUO, infiltration of inflammatory cells plays a key role in the initiation and development of kidney injury.^42^ Furthermore, tubular epithelial cells can produce TNF‐α, which stimulates the release of IL‐1β and CCL2 and has a prominent role in glomerular inflammation and fibrosis.^43^ On the contrary, fibrotic responses may also contribute to inflammation.^44^ In this way, UUO‐induced inflammation and renal fibrosis can influence each other.

We also investigated the fibrotic signalling pathways induced by UUO that may be affected by CHRFAM7A expression. Multiple signalling pathways are involved in the pathogenesis of renal fibrosis after UUO, including TGF‐β1/Smad3 and P38/MAPK.^45^, ^46^ Some studies show that TGF‐β1 plays a key role in the process of renal fibrosis and it can act on renal tubular epithelial cells, and inflammatory cells.^47^ TGF‐β1 stimulates fibroblast proliferation, EMT and synthesis of ECM that promotes the fibrosis in many organs.^13^, ^48^ TGF‐β1 can activate downstream mediators, specifically Smad3, which leads to gene expression changes involved in the pathogenesis of renal interstitial fibrosis.^49^ In fact, it has been reported that knockout of Smad3 can effectively prevent renal fibrosis in the mouse UUO injury model.^45^ Similarly, we observed that the mRNA and protein levels of TGF‐β1 and Smad2/3 in the kidney were significantly lower in the CHRFAM7A KI‐UUO group than that the WT‐UUO group. As discussed, TGF‐β1/Smad3 is an important signalling pathway related to the induction of EMT.^50^ Research has shown that CHRFAM7A is widely expressed in white blood cells and epithelial cells, which routinely undergo EMT‐like processes.^31^ In our in vitro experiments, we transfected a CHRFAM7A‐containing plasmid or empty vector plasmid into human renal tubular epithelial cells (HK‐2), and then induced EMT with recombinant human TGF‐β1. We found that mRNA expression of fibrosis‐related factors α‐SMA and FN‐1 in the CHRFAM7A + TGF‐β1 group was significantly reduced compared with the empty vector + TGF‐β1 group. We also observed that overexpression of human‐specific CHRFAM7A reduced the expression of fibrosis‐related factors induced by TGF‐β1 in HK‐2 cells. The expression of the CHRFAM7A gene prohibited the TGF‐β1/Smad2/3 signalling pathway. It also prevented TGF‐β1‐induced expression of mesenchymal markers N‐cadherin and vimentin. Together, our data show that the human‐specific CHRFAM7A gene may be able to down‐regulate the TGF‐β1/Smad2/3 signalling pathway in the kidney, delaying the progression of renal fibrosis caused by obstructive injury.

In summary, our experimental results show that the human‐specific CHRFAM7A gene can reduce renal fibrosis in mice with obstructive nephropathy by down‐regulating the TGF‐β1/Smad2/3 signalling pathway as well as inhibiting the release of inflammatory factors. Targeting the cholinergic anti‐inflammation pathway may be a promising protective factor for kidney structure and function after obstructive injury.

## AUTHOR CONTRIBUTIONS

**Bingru Zhou:** Conceptualization (equal); data curation (equal); formal analysis (equal); investigation (lead); resources (equal); writing – original draft (equal); writing – review and editing (equal). **Yudian Zhang:** Conceptualization (equal); data curation (equal); formal analysis (equal); investigation (supporting); resources (equal); supervision (supporting); writing – original draft (equal); writing – review and editing (supporting). **Xitong Dang:** Investigation (supporting); resources (equal); supervision (equal); writing – review and editing (equal). **Bowen Li:** Data curation (equal); formal analysis (equal); writing – review and editing (equal). **Hui Wang:** Data curation (equal); formal analysis (equal); writing – review and editing (equal). **Shu Gong:** Data curation (equal); writing – review and editing (equal). **Siwen Li:** Data curation (supporting); writing – review and editing (supporting). **Fanyin Meng:** Investigation (supporting); writing – review and editing (equal). **Juan Xing:** Formal analysis (supporting); writing – review and editing (supporting). **Tian Li:** Writing – review and editing (supporting). **Longfei He:** Formal analysis (supporting); writing – review and editing (supporting). **Ping Zou:** Conceptualization (lead); investigation (equal); resources (equal); supervision (equal); writing – original draft (equal); writing – review and editing (equal). **Ying Wan:** Conceptualization (lead); funding acquisition (lead); project administration (lead); resources (lead); supervision (lead); writing – review and editing (lead).

## CONFLICT OF INTEREST

The authors have declared that no conflicts of interest exist.

## Supporting information


Figures S1–S2.

Table S1.

## Data Availability

All data generated or analysed during this study are included in this article.
