# Supplementary material for: Up‐regulation of the human‐specific CHRFAM7A gene protects against renal fibrosis in mice with obstructive nephropathy
Source: J Cell Mol Med. 2022 Dec 7;27(1):52–65. doi: 10.1111/jcmm.17630 (PMC9806291; doi:10.1111/jcmm.17630)
Supplement: Supplementary file 1 — Figures S1–S2. [file JCMM-27-52-s002.pptx]

## Slide 1
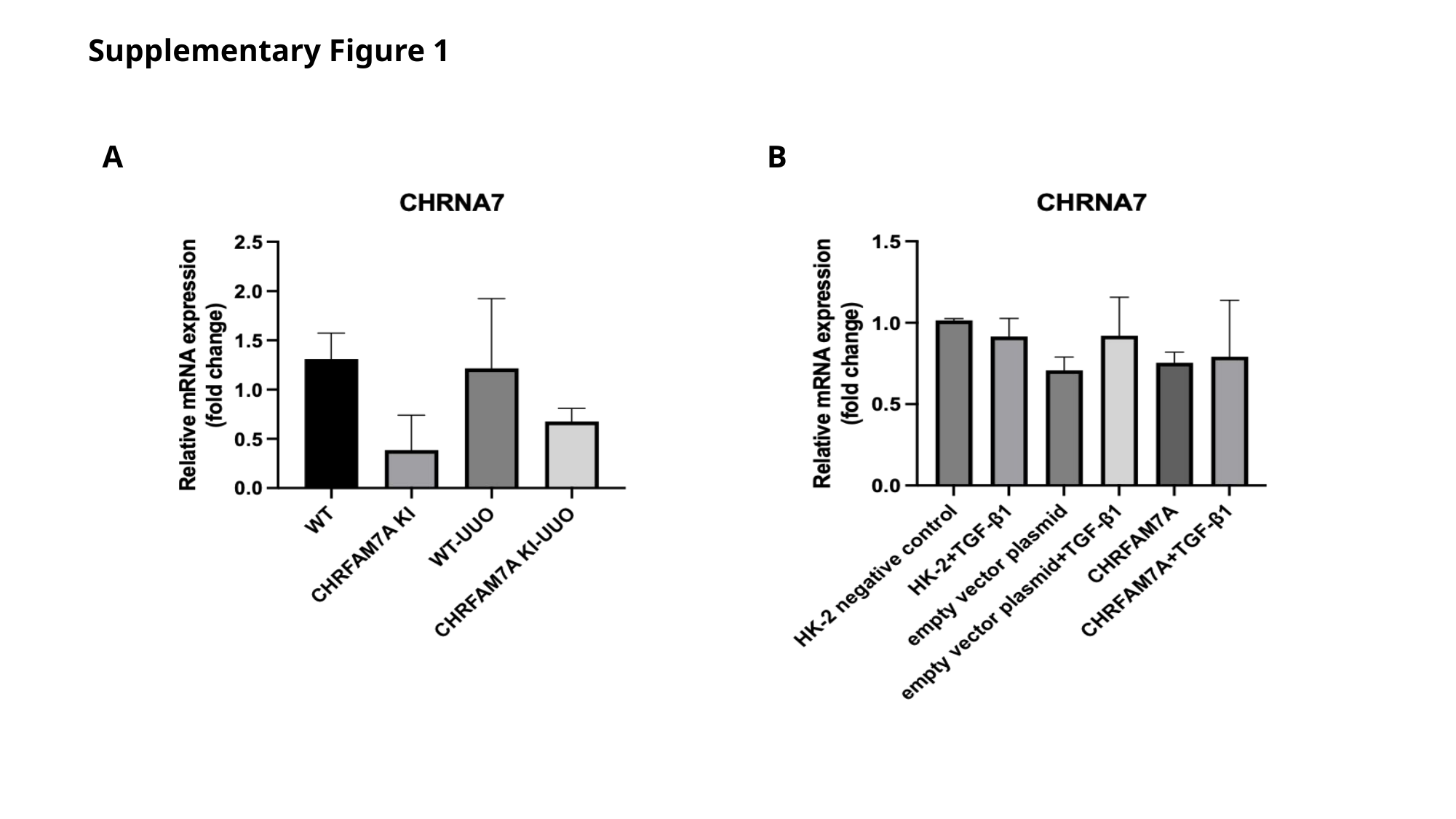

Supplementary Figure 1
A
B

## Slide 2
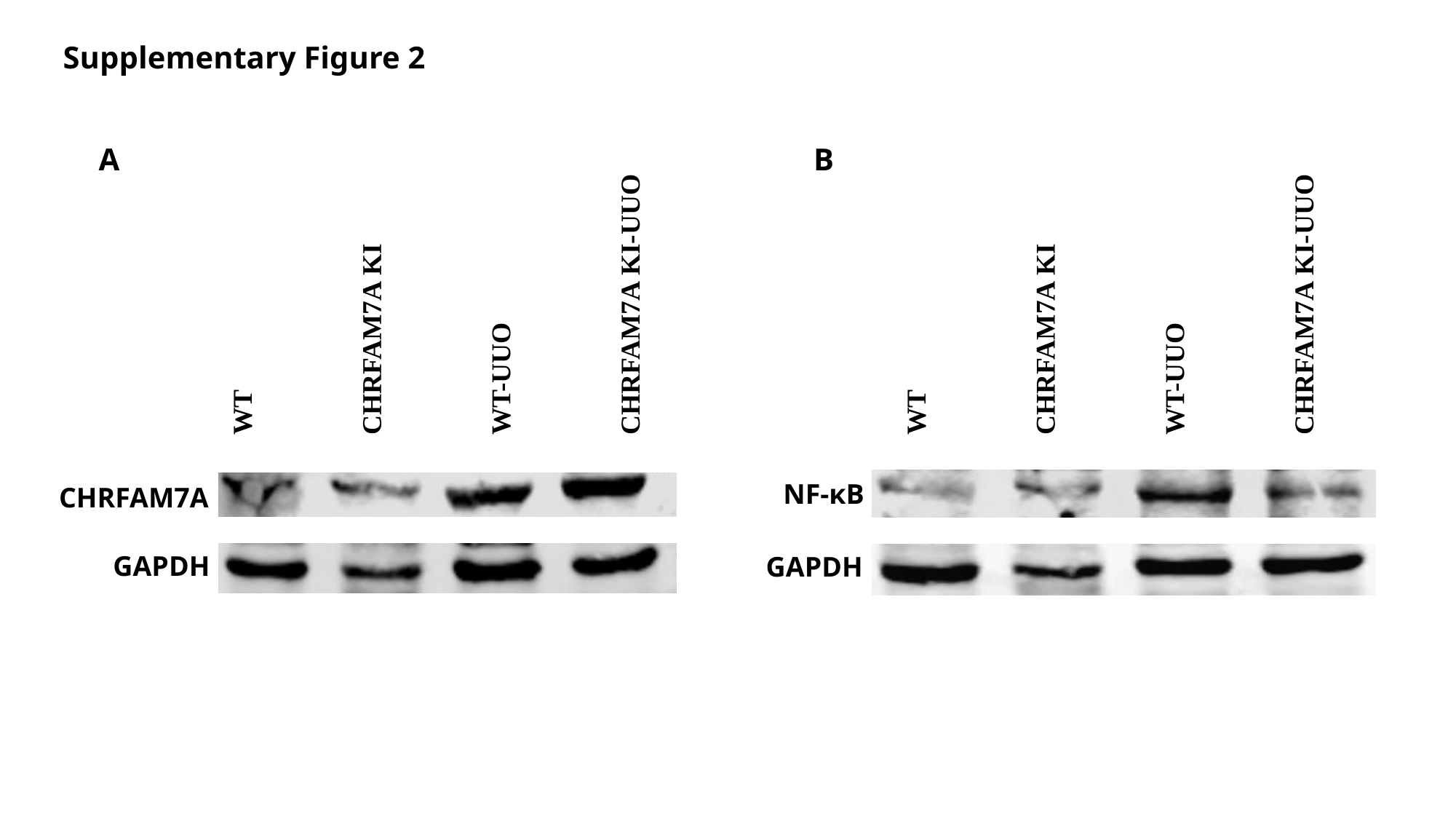

Supplementary Figure 2
A
B
CHRFAM7A KI-UUO
CHRFAM7A KI
WT-UUO
WT
CHRFAM7A
GAPDH
CHRFAM7A KI-UUO
CHRFAM7A KI
WT-UUO
WT
NF-κB
GAPDH

## Slide 3
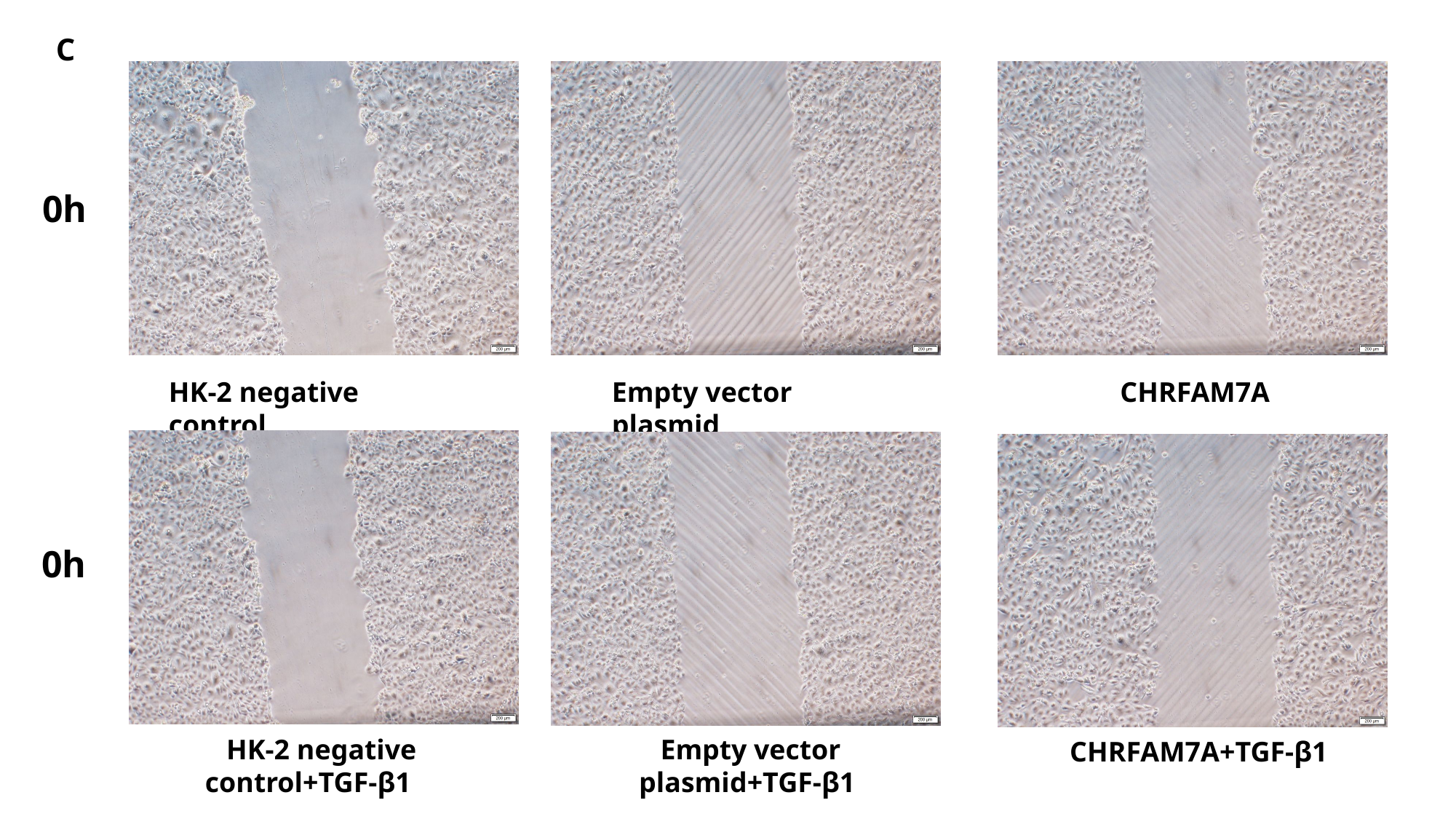

C
0h
Empty vector plasmid
HK-2 negative control
CHRFAM7A
0h
 HK-2 negative control+TGF-β1
 Empty vector plasmid+TGF-β1
CHRFAM7A+TGF-β1

## Slide 4
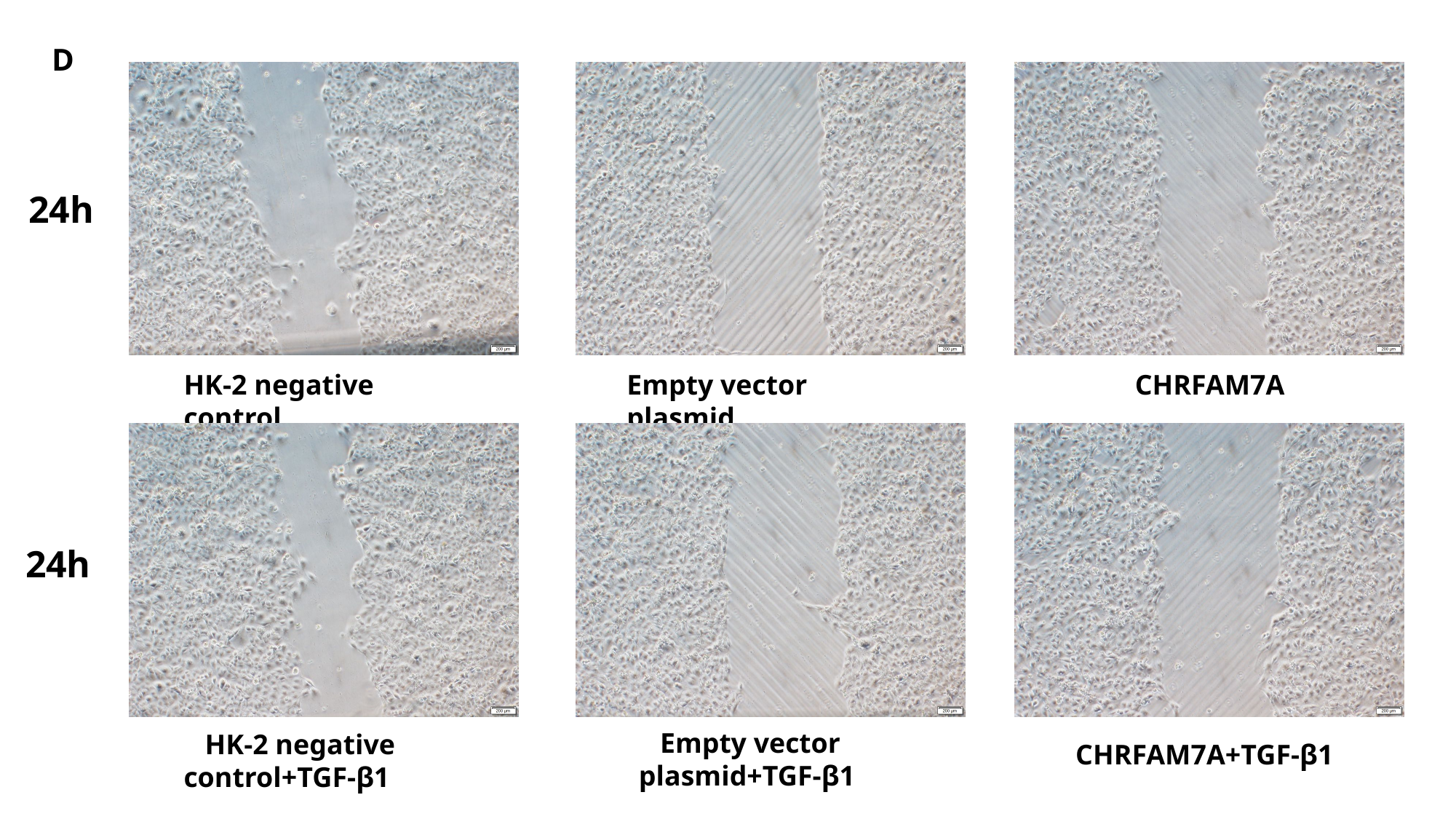

D
24h
Empty vector plasmid
HK-2 negative control
CHRFAM7A
24h
 Empty vector plasmid+TGF-β1
 HK-2 negative control+TGF-β1
CHRFAM7A+TGF-β1
