# Supplementary material for: Up‐regulation of the human‐specific CHRFAM7A gene protects against renal fibrosis in mice with obstructive nephropathy
Source: J Cell Mol Med. 2022 Dec 7;27(1):52–65. doi: 10.1111/jcmm.17630 (PMC9806291; doi:10.1111/jcmm.17630)
Supplement: Supplementary file 2 — Table S1. [file JCMM-27-52-s001.doc]

**Supplementary Table 1 List of real-time PCR primers used**

| **Gene** | **Species** | **Refseq** | **Primer sequence** |
| --- | --- | --- | --- |
| ACTA2(α-SMA) | Mouse | NM_007392 | Forward primer, 5’-TCGCTGGTGATGATGCT-3’  Reverse primer, 5’-TGGTGATGATGCCGTGT -3’ |
| CCL2 | Mouse | NM_011333 | Forward primer, 5’-AGGTCCCTGTCATGCTTCT-3’  Reverse primer, 5’ -GCTGCTGGTGATCCTCTT-3’ |
| CD206 | Mouse | [NM_008625.2](https://www.ncbi.nlm.nih.gov/nuccore/NM_008625.2) | Forward primer, 5’-TCCCTGTCTCTGTTCAGCTATTG-3’  Reverse primer, 5’ CGTCTGAACTGAGATGGCACTTA-3’ |
| CHRNA7 | Mouse | [NM_007390.3](https://www.ncbi.nlm.nih.gov/nuccore/NM_007390.3) | Forward primer, 5’-ACAGTGCAGATGAACGCTTTG-3’  Forward primer, 5’-GAGCTCTTGAATATGCCTGGA-3’ |
| Fn-1 | Mouse | NM_010233 | Forward primer, 5’-CGTCATTGCCCTGAAGA-3’  Reverse primer, 5’-GAAGATTGGGGTGTGGAA-3’ |
| FIZZ1 | Mouse | [NM_020509.4](https://www.ncbi.nlm.nih.gov/nuccore/NM_020509.4) | Forward primer, 5’-CCTCCACTGTAACGAAGACTCTC-3’  Reverse primer, 5’-CTCCCAAGATCCACAGGCAAAG-3’ |
| GAPDH | Mouse | NM_008084 | Forward primer, 5’-TGTTTCCTCGTCCCGTAGA-3’  Reverse primer, 5’-ATCTCCACTTTGCCACTGC-3’ |
| KIM-1 | Mouse | NM_001166632.1 | Forward primer, 5’-CTAAGCGTGGTTGCCTTC-3’  Reverse primer, 5’-TGTCTTCAGCTCGGGAAT-3’ |
| IL-1β | Mouse | NM_008361 | Forward primer, 5’-AGTTGACGGACCCCAAA-3’  Reverse primer, 5’-TCTTGTTGATGTGCTGCTG-3’ |
| IL-6 | Mouse | NM_031168 | Forward primer, 5’-ACAGAAGGAGTGGCTAAGGA-3’  Reverse primer, 5’-AGGCATAACGCACTAGGTTT-3’ |
| Smad 3 | Mouse | NM_001252481 | Forward primer, 5’-TGAGGTTTGGAAGCTGAGA-3’  Reverse primer, 5’-AGTTTGCTGTGGCAATCC -3’ |
| TGF-β1 | Mouse | NM_011577 | Forward primer, 5’-GCAACAATTCCTGGCGTTA-3’  Reverse primer, 5’-TTCCGTCTCCTTGGTTCAG-3’ |
| TNF-α | Mouse | NM_013963 | Forward primer, 5’- CGCTGAGGTCAATCTGC-3’  Reverse primer, 5’- GGCTGGGTAGAGAATGGA-3’ |
| ACTA2(α-SMA) | Human | NM_001141945 | Forward primer, 5’-GTTACGAGTTGCCTGATGG-3’  Reverse primer, 5’- AGGTGGTTTCATGGATGC-3’ |
| CHRFAM7A | Human | [NM_139320.2](https://www.ncbi.nlm.nih.gov/nuccore/NM_139320.2) | Forward primer,5′-CAGATATCCAATGGGCGTGGA-3′  Forward primer,5′-TGGAATGTGTGGCGTCAAAGCG-3′ |
| CHRNA7 | Human | [NM_000746.6](https://www.ncbi.nlm.nih.gov/nuccore/NM_000746.6) | Forward primer, 5’-GCTGCAAATGTCTTGGACAG-3’  Reverse primer, 5’- TTTCCAAATCTGGCCATCTG-3’ |
| E-cadherin | Human | [NM_001317184.2](https://www.ncbi.nlm.nih.gov/nuccore/NM_001317184.2) | Forward primer, 5’-GTCCTGGGCAGAGTGAATT-3’  Reverse primer, 5’-ACACCATCTGTGCCCACTT-3‘ |
| FN-1 | Human | NM_002026 | Forward primer, 5’-ATTCTGTAGGCCGTTGGA-3’  Reverse primer, 5’- TACTGCTGGATGCTGATGA-3’ |
| GAPDH | Human | NM_001256799 | Forward primer, 5’-GGGGCTCTCCAGAACATC-3’  Reverse primer, 5’-TGACACGTTGGCAGTGG-3’ |
| N-cadherin | Human | [NM_001308176.2](https://www.ncbi.nlm.nih.gov/nuccore/NM_001308176.2) | Forward primer, 5’-GGTGGAGGAGAAGAAGACCA-3’  Reverse primer, 5’-TTCATCCATTCGTCGGATTC-3’ |
| TGF-β1 | Human | NM_000660 | Forward primer, 5’-ACCACACCAGCCCTGTTC-3’  Reverse primer, 5’- CGTCAGCACCAGTAGCCA-3’ |
| Vimentin | Human | [NM_003380.5](https://www.ncbi.nlm.nih.gov/nuccore/NM_003380.5) | Forward primer, 5’-CTCCGGGAGAAATTGCAG-3’  Reverse primer, 5’- GCCAGAGACGCATTGTCAAC-3’ |
